# Implementing an intervention to improve leadership/management of public healthcare services in the Free State Province, South Africa: lessons learned

**DOI:** 10.4314/ahs.v23i1.49

**Published:** 2023-03

**Authors:** Benjamin Malakoane, Perpetual Chikobvu, James Christoffel Heunis, Nanteza Gladys Kigozi, Willem Hendrik Kruger

**Affiliations:** 1 Department of Community Health, University of the Free State, PO Box 339, Bloemfontein, 9300; 2 Centre for Health Systems Research & Development, University of the Free State, PO Box 339, Bloemfontein, 9300

**Keywords:** Public health system, intervention, implementation, systems theory, balanced scorecard, leadership/management

## Abstract

**Background:**

Based on the World Health Organization's health systems strengthening framework, the Health Systems Governance and Accountability (HSGA) intervention to strengthen public health leadership/management, service integration and outcomes was developed in the Free State.

**Objectives:**

This study describes the process to implement and measure the effects of the HSGA intervention for system-wide improvement of leadership/management under routine conditions in a resource-constrained setting.

**Methods:**

Based on normalisation process theory, participatory discussions were held with health managers, staff and local stakeholders to attain buy-in. Evaluation of the implementation process considered progress in improving leadership/management through application of the Balanced Scorecard (BSC). All provincial reporting units were assessed during 2014/15 and again during 2015/16.

**Results:**

The mean scores on three BSC perspectives improved statistically significantly from 2014/15 to 2015/16: customer (*p*=0.0085), internal business processes (*p=*0.0008) and finance (*p*=0.0001). Overall leadership/management also improved significantly (*p*=0.0007).

**Conclusion:**

Improvement in leadership/management resulting from implementation of the HSGA intervention was observed during the two years under study. From this experience, successful implementation of a health systems strengthening intervention hinges on a participatory design, appropriate use of theory, as well as application of an evaluation approach to assess the success of implementation.

## Background

It is increasingly recognised that the implementation process or ‘how-to’ of health interventions to improve leadership/management of public health services is as important to document and understand as the features and functions of the interventions themselves^1^. However, implementation processes are often poorly reported with inadequate description of context and incomplete evidence on how the intervention was promoted and implemented (‘or not’) in specific settings^2^. Insufficient detail on the implementation process and the fit between the intervention and the context may hinder replication and scale-up of interventions elsewhere^1^.

According to Nilsen^3^, implementation science theoretical approaches serve to describe and/or guide processes to change practice (process models) and/or explain what influences implementation outcomes (determinant frameworks). Since the determinants of intervention outcomes may be too generic to guide implementation processes, most determinant frameworks provide little ‘how-to’ support for carrying out implementation endeavours. Contrarily, process models focus on providing ‘how-to’ information.

The process followed by the Free State Department of Health (FSDoH) to develop the Health Systems Governance and Accountability (HSGA) model and policy^4^ (hereafter HSGA intervention) – broadly based on the WHO's health system building block framework^5^,^6^ is detailed elsewhere. The Free State Province experienced rising disease burdens and inadequate resource allocation. Across the facilities (hospitals, districts and clinics) there were high vacancy rates and as a result there was ineffective programme implementation with resultant poor outcomes. The province experienced leadership challenges as fragmentation of service delivery continued unabated. The HSGA intervention focused on the bigger problem of fragmentation of services across the whole public health system as opposed to only the lower programme or facility levels. A systems approach was used to understand how the multiple elements involved in patient care independently and interdependently interacted with each other during the implementation process^7^. The WHO health systems strengthening (‘Building Block’) framework was employed as a platform to help the intervention implementers to address the complexity of the Free State public health system and the interactions amongst its various components.

This study describes the ‘how-to’ of an intervention to improve leadership/management of public health services using the HSGA approach to improve integration and service-delivery outcomes through implementation of a ‘whole-system’ intervention in a public health setting with limited resources and a high burden of disease^8^,^9^,^10^. Given the importance of leadership/management in health systems strengthening^5^,^6^, the paper focuses on the processes followed in the implementation of the HSGA intervention to improve leadership/management capacity by application of Kaplan and Norton's^11^ Balanced Scorecard (BSC) performance-monitoring approach.

One of nine provinces in South Africa, the Free State accommodates 5.1% of the public health sector dependant population of whom more than 80% are African^12^ and historically and socioeconomically disadvantaged due to apartheid spatial and homeland planning^13^, inequality in public funding allocation^14^, social exclusion and segregated access to public sector amenities^13^,^15^. In 2015/16, primary health care (PHC) in the Free State was provided by 211 fixed PHC clinics, ten community health centres (CHCs) and numerous mobile clinics^8^ across five districts, i.e., Mangaung, Lejweleputswa, Fezile Dabi, Thabo Mofutsanyana and Xhariep. Hospital services included 24 district, four regional, one specialised psychiatric, one tertiary and one central hospital. The PHC clinics, CHCs and hospitals are respectively responsible for primary, secondary, and tertiary care services. District health managers (DCSTs) are responsible for planning and monitoring of disease control programme implementation within their districts, with District Clinical Specialist Teams providing supportive supervision, clinical governance, and attending to health systems and logistics, staff development and user-related considerations^8^,^16^.

## Methods

A participatory design was used to implement the intervention, with users being closely engaged in the design and development of the implementation plan, as well as its implementation or operationalisation. The HSGA intervention was implemented across the entire public healthcare system and encompassed broad stakeholder (inter alia managers, non-governmental organisations, traditional healers) and community involvement through meetings and feedback sessions. Starting in February 2014, the incremental implementation of the HSGA intervention and concomitant use of the BSC to monitor and evaluate compliance to policy and processes and the impact of changes, were used to ensure sustainable implementation^17^.

In the current study, the BSC was used to monitor and evaluate service integration and performance by collecting, assessing and reporting on data collected during routine service delivery processes, leadership/management performance assessments and in implementing health system reforms. The BSC requires managers to look at 'business from the i) customer perspective: ‘how do customers see us?’; ii) internal business processes perspective: ‘what must we excel at?’; iii) organisational capacity perspective: ‘can we continue to improve and create value?’; and iv) financial perspective: ‘how do we look to shareholders?’ Participants (managers) were required to assess overall leadership/management performance across these four perspectives and to note how leadership was displayed by various functionaries in respect of each perspective. The BSC was further used to assess change in the leadership/management provided by the executive leaders and the health programme managers representing all 50 reporting units of the FSDoH.

The scores for each of the five BSC perspectives were measured as follows: i) the customer perspective was measured using four activities; ii) the internal business processes perspective using 13 activities; iii) the organisational capacity perspective using four activities; and iv) the financial perspective using four activities. A total of 25 activities that were used to measure the leadership goal. During the assessment, achievement of each activity was given a category of ‘1’ if the activity was supported with sufficient evidence, otherwise ‘0’ if the activity was not achieved and was not supported with sufficient evidence. The BSC score for each reporting unit was calculated as the sum of all the categories achieved divided by 25 and multiplied by 100. This score ranged between zero and 100 percent with 100% indicating achievement of all the activities and 0% indicating non-achievement of any activity. Data from the BSC assessments was analysed by means of frequency distributions and percentages for discrete variables and measures of central tendency for continuous variables. The differences in the BSC scores recorded for the financial years 2014/15 and 2015/16 respectively were measured through chi-square tests at a 0.05 significance level.

**Figure 1:**
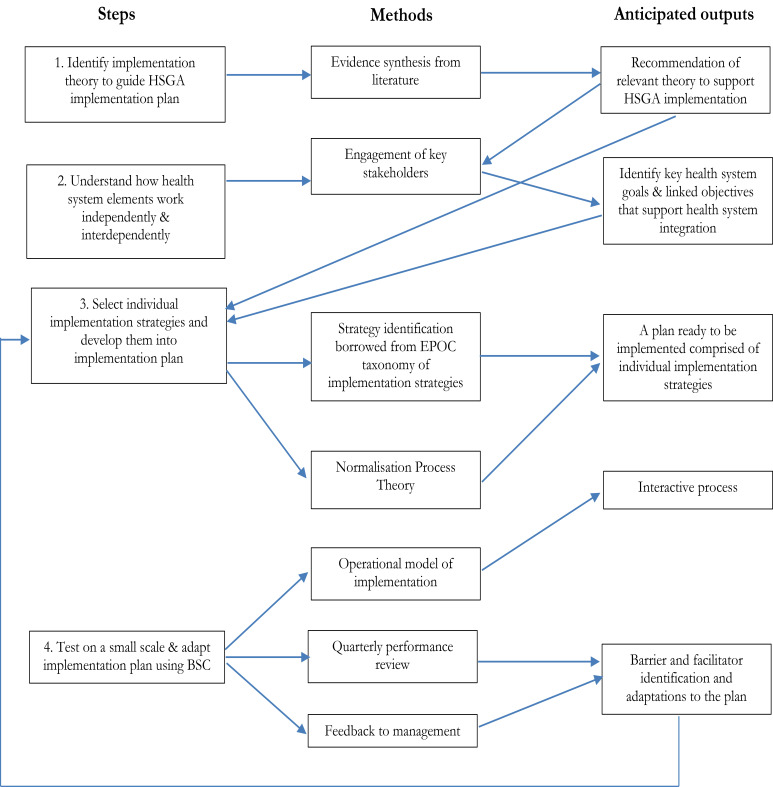
Development of the HSGA intervention implementation plan

Similar to Ross et al.'s^1^ approach, the delivery of the intervention was guided by the steps, methods and anticipated outputs outlined in Figure 1.

**Figure 2:**
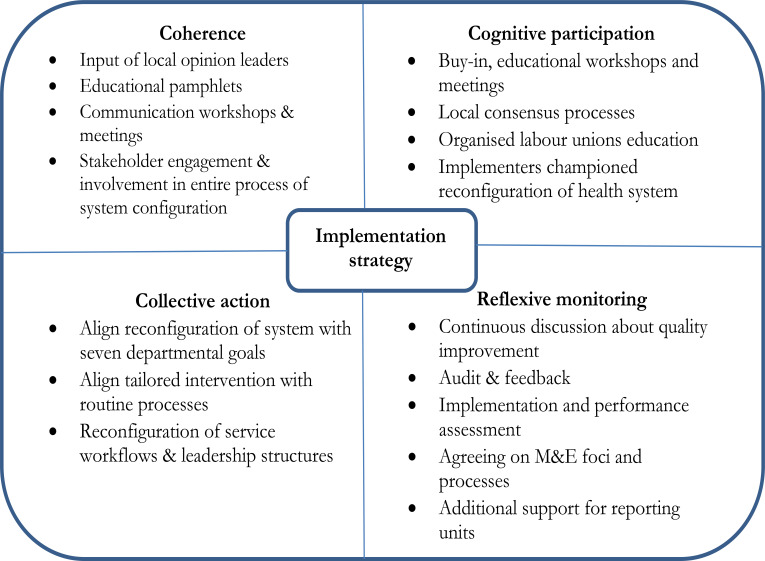
Implementation strategies selected to target constructs of Normalisation Process Theory

With reference to Step 1, to ‘identify the implementation theory to guide implementation plan for HSGA,’ the Normalisation Process Theory (NPT) (Figure 2) was adopted, to guide the development of the implementation plan. The NPT can be used to inform implementation and integration of complex interventions into routine health care^1^,^17^,^18^. The four main components of the NPT namely ‘coherence’, ‘cognitive participation’, ‘collective action’, and ‘reflexive monitoring’ were considered in the implementation of the HSGA intervention. Normalisation was facilitated by actively involving healthcare managers, frontline healthcare workers and community members to understand and facilitate the implementation of the HSGA intervention processes into routine management and practice.

The use of this theory enabled consideration of the barriers and facilitators identified during the situational appraisal^8^ and the selection of appropriate implementation strategies. The NPT as applied and recommended by May et al.^17^ brings practice to action including embedding practices into everyday work, as well as service integration where practices are reproduced and sustained within the operational matrices of an organisation. The selection of change strategies was done based on the Cochrane Effective Practice and Organisation of Care (EPOC) taxonomy of implementation strategies^19^. Since the HSGA intervention was designed to change the organisational leadership/management culture aimed at integration of service delivery and improving performance, we focused on the category of interventions targeted at the entire organisation.

Regarding Step 2, to ‘understand how health system components operate independently and interdependently,’ meetings with the senior management of the FSDoH were convened to develop a strategy to implement the changes. The intended interventions and improvements were also discussed with local communities, traditional leaders, health practitioner organisations and thought leaders during a health ‘indaba’ (collective discussion or meeting) to incorporate their inputs, and obtain their buy-in and support.

In respect to Step 3, to ‘select individual implementation strategies and develop them into an implementation plan,’ the annual performance plan was reviewed, the annual targets aligned with the change mechanisms, and the functions of different reporting units changed to enable monitoring and evaluation of performance through the BSC approach. Functionaries were expected to work towards achievement of the objectives linked to the goals and to ensure that there was an audit trail of evidence for assessment and accountability purposes.

The delivery of the change mechanism included various actions to enhance integration and improve the outcomes of public health service delivery. In respect to service delivery, the one-stop service-rendering plan had to be strengthened and the services re-organised accordingly - specifically to enable PHC facilities to offer a comprehensive package of PHC services^20^. Medicines, vaccines and consumables were declared as ‘non-negotiable’ items in order to enforce continuous monitoring and evaluation of their availability in all healthcare facilities. Procedures for referral covering patient movement from the home setting to the local fixed or mobile clinic up the referral ladder to the tertiary and central levels of care were implemented. Ambulance services were restructured after consultation with the operators. Route mapping was conducted to decant patients more effectively to identified central points. The DCSTs were responsible for performing regular facility visits to observe and address weaknesses in the coordination of patient flows, referrals and treatment processes.

Regarding leadership/management, policies and procedures were reviewed with the purpose of integrating various functions and doing away with a ‘silo’ approach. New and reviewed interlinked policies and procedures were implemented, and change management processes were collaboratively implemented to obtain buy-in from all levels of managers, as well as community members and other stakeholders. Specific managers were assigned responsibilities to act as change agents (‘champions’) within their functional lines and to foster integrative functioning.

Regarding Step 4, to ‘test the implementation plan and refine on small scale,’ a phased- in approach was adopted where pre-determined objectives were incrementally evaluated on a district-by-district basis and refinements made accordingly. This cautious approach is corroborated by Wight et al.'s^21^ view that since repeated testing and monitoring and evaluation is required, large-scale integration of services and policy changes are difficult to pilot for a huge public health service. This is especially the case where the intervention is a novel idea that has not been implemented elsewhere.

The implementation plan was thus informed by literature review, theory of implementation, analysis of the departmental leadership's feedback, and the use of the information collected during the development of the intervention. The implementation strategies were selected in line with the constructs of NPT and thus increased the likelihood of implementation. It was hypothesised that healthcare managers and workers would lead the implementation demonstrating that they understood the purpose of the intervention (‘coherence’), would be prepared to invest time and energy into it (‘cognitive participation’), would feel that the intervention fitted well with their current work (‘collective action’), and would perceive the intervention to be worthwhile (‘reflexive monitoring’). Operationalisation of these constructs and strategies were targeted for the HSGA intervention within the implementation plan (Table 1).

**Table 1:** Views related to Goal 1 Leadership/governance

		Yes n (%)	No n (%)	DNK n (%)
**HMs** **(n=147)**	• Health sector reforms implemented	67 (45.58)	10 (6.80)	70 (47.62)
	• District health plans implemented	87 (59.18)	17 (11.56)	43 (29.25)
	• Hospitals service delivery plans implemented	61 (41.50)	14 (9.52)	72 (48.98)
	• Clinic management structures in place and functional	65 (44.22)	29 (19.73)	50 (34.01)
	• Corporate office influenced district health plans	33 (22.45)	7 (4.76)	107 (72.79)
	• Corporate office influenced hospital health plans	28 (19.05)	8 (5.44)	111 (75.51)

**CRs** **(n=78)**	• Health sector reforms implemented	46 (58.97)	14 (17.95)	18 (23.08)

HM, health manager; CR, community representative, DNK, do not know

During the implementation of the HSGA intervention, 32 actions were implemented to improve healthcare service integration and performance. As shown in Table 2, these actions covered the seven goals of the health department, namely, to improve: i) leadership/governance, ii) financial management, iii) workforce management, iv) PHC re-engineering, v) infrastructure management, vi) HIMS and vii) referral and ‘whole- system’ interventions (Table 2).

**Table 2:** Views related to Goal 2 Financial management

		All n (%)	Most n (%)	Some n (%)	None n (%)	DNK n (%)
**HMs** **(n=147)**	• Hospitals charged fees for services	57 (38.78)	19 (12.93)	31 (21.09)	9 (6.12)	31 (21.09)
	• Records of accounting procedures in place	37 (25.17)	15 (10.20)	6 (4.08)	7 (4.76)	82 (55.78)
	• Periodic audits conducted	31 (21.09)	9 (6.12)	8 (5.44)	8 (5.44)	91 (61.90)
	• Monthly financial reports delivered	41 (27.89)	11 (7.48)	4 (2.72)	6 (4.08)	85 (57.82)
	• Expenditure reports in use	44 (29.93)	17 (11.56)	10 (6.80)	6 (4.08)	70 (47.62)
	• Revenue reports in use	43 (29.25)	17 (11.56)	11 (7.48)	5 (3.40)	71 (48.30)
	• Record of accounting procedures in use	33 (22.45)	21 (14.29)	11 (7.48)	5 (3.40)	77 (52.38)
	• Periodic audit reports in use	32 (21.77)	8 (5.44)	16 (10.88)	7 (4.76)	84 (57.14)
	• Monthly financial reports in use	46 (31.29)	13 (8.84)	8 (5.44)	5 (3.40)	75 (51.02)
**CRs** **(n=78)**	• Hospitals charged fees for services	22 (28.21)	11 (14.10)	17 (21.79)	16 (20.51)	12 (15.38)
	• Morale of the staff always checked	26 (33.33)	13 (16.67)	11 (14.0)	13 (16.67)	15 (19.23)

CR, community representative; DNK, do not know; HM, health manager; n, number

In addition, quarterly performance assessments with feedback were conducted and recorded by the executive leadership and a panel of assessors. These assessments were based on tangible evidence of performance, assessed and checked for relevance, validity and reliability. The intervention was continuously refined through a documented data feedback mechanism during the quarterly performance assessments. Reports were analysed and feedback was provided to the management teams to enable refinement. Policy uncertainties and other weaknesses were identified, and corrective action taken. During this process, certain objectives were chosen for measurement together with key indicators related to each objective to verify the outcomes of health systems strengthening and integration.

## Results

As shown in Table 3, all 50 reporting units of the FSDoH were assessed during the first year of the study (2014/15) and 44 during the second year (2015/16). The reduction in the number of reporting units in the second year was due to complexing of smaller units into bigger units reporting to a single manager. Thus, no facilities or services included in the first year were excluded in the complexed configuration of facilities or services in the second year. The median scores on the customer perspective were between zero and 50.0% in the first year and 25.0% and 50.0% in the second year, which was not statistically significant (p=0.078). However, the mean score improved from 23.0% in the first year to 38.6% in the second year, which was statistically significant (*p*=0.0085).

**Table 3:** Views related to Goal 3 Workforce management

		All staff n(%)	Most staff n (%)	Some staff n (%)	No staff n (%)	DNK n (%)
**HMs** **(n=146)**	• Districts had job descriptions	21 (14.38)	40 (27.40)	53 (36.30)	8 (5.48)	24 (16.44)
	• Districts had training plans	14 (9.59)	38 (26.03)	59 (40.41)	11 (7.53)	24 (16.44)
	• Districts had career plans	11 (7.53)	35 (23.97)	61 (41.78)	14 (9.59)	25 (17.12)
	• Districts had staff assessment systems	15 (10.27)	37 (25.34)	62 (42.47)	10 (6.85)	22 (15.07)
	• Districts had staff rotation systems	11 (7.53)	31 (21.23)	67 (45.89)	12 (8.22)	25 (17.12)
	• Hospitals had job descriptions	10 (6.85)	39 (26.71)	63 (43.15)	4 (2.74)	30 (20.55)
	• Hospitals had training plans	7 (4.79)	37 (25.34)	67 (45.89)	8 (5.48)	27 (18.49)
	• Hospitals had career plans	7 (4.79)	32 (21.92)	70 (47.95)	9 (6.16)	28 (19.18)
	• Hospitals had staff assessment	6 (4.11)	35 (23.97)	73 (50.00)	6 (4.11)	26 (17.81)
	• Hospitals had staff rotation systems	5 (3.42)	37 (25.34)	74 (50.68)	4 (2.74)	26 (17.81)

				**Yes** **n (%)**	**No** **n (%)**	**DNK** **n (%)**
	• Hospitals had up-to-date staff status reports			33 (22.60)	6 (4.11)	107 (73.29)
	• Performance agreements of all the staff in HM's institution are up-to-date			59 (40.41)	21 (14.38)	66 (45.21)
	• Morale of staff in HM's institution is assessed			48 (32.88)	37 (25.34)	61 (41.78)

		**All staff** **n (%)**	**Most staff** **n (%)**	**Some staff** **n (%)**	**No staff** **n (%)**	**DNK** **n (%)**

**CRs** **(n=78)**	• Morale of the staff is always checked	26 (33.33)	13 (16.67)	11 (14.10)	13 (16.67)	15 (19.23)

CR, community representative; DNK, do not know; HM, health manager; n, number

The median scores on the internal business processes perspective were between zero and 30.8% in the first year and between 7.8% and 61.5% during the second year, which was statistically significant (*p*=0.016). The mean score increased from 18.3% in the first year to 36.9% in the second year, which was also statistically significant (*p*=0.0008).

Although the median score for the organisational capacity perspective during the second year was higher than in the first year of study (0.0% vs 25.0%), this difference was not statistically significant (*p*=0.220). The difference in the mean score from the first to the second year (19.0% vs 38.1%) was also not statistically significant (*p*=0.0653). The median score on the finance perspective increased from 25.0% in the first year to 54.0% in the second year which was statistically significant (*p*=0.007), as was the difference in the mean score that increased from 28.5% in the first year to 58.0% in the second year (*p*=0.0001).

Considering overall leadership/management, half of the median scores were between zero and 36% in the first year and between 20% and 60% in the second year, which was a statistically significant (*p*=0.016) improvement. The mean score improved from 20.8% in the first year to 38.2%, which likewise was statistically significant (*p*=0.0007). Authors have suggested that the effect size of 0.2 could be considered as ‘small,’ 0.5 as ‘medium’ and 0.8 as ‘large’ changes^22^,^23^. In the current study, the effect size for the measured perspectives ranged from 0.607 to 0.960. This demonstrated that there were medium to large changes in the leadership goal perspectives with an effect change of 0.734 for the overall leadership goal (Table 3).

Table 4 shows a sensitivity analysis when considering only the 44 reporting units that were assessed in both financial years excluding those that were changed or merged. This analysis was done to assess whether there was any substantial difference in the scores when the six sites were excluded from the baseline analysis. The effect sizes of the sensitivity analysis (Table 4) were similar to those presented in Table 3. There were still medium to large positive changes in the BSC scores related to the leadership/management goal (Table 4).

**Table 4:** Views related to Goal 4 PHC re-engineering

		Completely n (%)	Partially n (%)	Not at n (%)	DNK n (%)
	**Views on extent to which PHC services were integrated into** **system**				

**HMs (n=146)**	• School health teams	42 (28.97)	62 (42.76)	5 (3.45)	36 (24.83)
	• Outreach services	42 (28.97)	63 (43.15)	8 (5.48)	33 (22.60)
	• Healthy lifestyle promotion	38 (26.03)	60 (41.10)	13 (8.90)	35 (23.97)
	• WBPHCOTs	35 (23.97)	62 (42.47)	11 (7.53)	38 (26.03)
	• DCST services	32 (21.92)	49 (33.56)	19 (13.01)	46 (31.51)
	• Contracted general practitioners	23 (15.75)	57 (39.04)	15 (10.27)	51 (34.93)
	• Development partners	39 (26.71)	59 (40.41)	6 (4.11)	42 (28.77)

			**Yes** **n (%)**	**No** **n (%)**	**DNK** **n (%)**
	**Views whether communities were actively involved in implementation of** **health reforms**				

**CRs (n=78)**	• Ideal clinic		56 (71.79)	6 (7.69)	16 (20.51)
	• HSGA intervention		42 (53.85)	7 (8.97)	29 (37.18)
	• BSC performance-monitoring tool		37 (47.44)	5 (6.41)	36 (46.15)
	• One patient-One file		51 (65.38)	3 (3.85)	24 (30.77)

BSC, Balanced Scorecard; CR, community representative; DCST, District Clinical Specialist Team; DNK, do not know; HM, health manager; HSGA, Health System Governance and Accountability; n, number; PHC, primary health care; WBPHCOT, ward-based PHC outreach team

Table 5 compares the median scores on the four BSC perspectives between the two years for different categories of reporting units, i.e., hospitals, districts and programmes. There were statistically significant (*p*<0.05) improvements in all the perspectives in median scores for hospitals between 2014/15 and 2015/16, except for the organisational capacity perspective (*p*=0.0944).

**Table 5:** Views related to Goal 5 Infrastructure management and Goal 6 Health Information Management System

				Yes n (%)	Partially n (%)	No n (%)	DNK n (%)
Infrastructure management	HMs (n=146)	• Participated in meetings on strategic infrastructure planning		22 (15.07)	22 (15.07)	68 (46.58)	34 (23.29)
		• Availability of equipment met expectations		15 (11.81)	31 (24.41)	68 (53.54)	13 (10.24)
		• Infrastructure maintenance met expectations		8 (5.48)	19 (13.01)	85 (58.22)	34 (23.29)

			VN n (%)	Neg n (%)	SN n (%)	NN n (%)	DNK n (%)

		• Influence of unavailability of equipment on health system performance	86 (58.90)	17 (11.64)	11 (7.53)	6 (4.11)	26 (17.81)
		• Influence of lack of maintenance on health system performance	87 (59.59)	15 (10.59)	13 (8.90)	2 (1.37)	29 (19.86)

				Yes n (%)	Partially n (%)	No n (%)	DNK n (%)

	CRs (n=78)	• Participated in meetings on strategic infrastructure planning		25 (32.05)	18 (23.08)	25 (32.05)	10 (12.82)
		• Availability of equipment met expectations		17 (21.79)	15 (19.23)	45 (57.69)	1 (1.28)
		• Infrastructure maintenance met expectations		20 (25.64)	12 (15.38)	41 (52.56)	5 (6.41)

			VN n (%)	Neg n (%)	SN n (%)	NN n (%)	DNK n (%)
		• Influence of unavailability of equipment on health system performance	22 (28.20)	29 (37.18)	16 (20.51)	4 (5.12)	7 (8.97)
		• Influence of lack maintenance on health system performance	17 (21.79)	27 (34.62)	14 (17.95)	4 (5.13)	16 (20.51)

					Yes n (%)	No n (%)	DNK n (%)

Health information management system	HMs (n=147)	• Whether possible based on the DHIS to indicate five diseases with the highest consultation rates in all districts			78 (53.06)	12 (8.16)	57 (38.78)
		• Whether possible based on the DHIS to indicate five diseases with the highest consultation rates in HM's district			80 (54.79)	13 (8.90)	53 (36.30)
		• Frequency of statistics form shortages at facilities	11 (7.59)	18 (12.41)	47 (32.41)	26 (17.93)	43 (29.66)
		• Frequency of submission of data to the DHIS by facilities	96 (66.21)	7 (4.83)	3 (2.07)	0 (0)	39 (26.90)
		• Frequency of analysis of statistics for decisionmaking by facility staff	40 (27.59)	34 (23.45)	24 (16.55)	5 (3.45)	42 (28.97)
		• Frequency of feedback reports to facilities by district-level staff	40 (27.59)	29 (20.00)	42 (28.97)	13 (8.97)	5 (3.45)
		• Frequency of use of health activity monitoring mechanisms	24 (16.55)	28 (19.31)	36 (24.83)	54 (37.24)	45 (31.03)
		• Frequency of submission of reports to the DHIS by traditional healers	6 (4.14)	6 (4.14)	18 (12.41)	5 (3.45)	84 (57.93)
		• Frequency of submission of reports to the DHIS by NGOs	27 (18.62)	20 (13.79)	11 (7.59)	17 (11.72)	80 (55.17)
		• Frequency of submission of reports to the DHIS by FBOs	8 (5.52)	12 (8.28)	7 (4.83)	24 (16.55)	94 (64.83)

					Yes n (%)	No n (%)	DNK n (%)

		• Whether possible to indicate five diseases with the highest consultation rates in CR's district			50 (64.10)	9 (11.54)	19 (24.36)

				Always n (%)	Mostly n (%)	Seldom n (%)	Never n (%)
		• Frequency of statistics form shortag es at facilities		11 (14.10)	19 (24.36)	25 (32.05)	23 (29.49)
		• Frequency of submission of data to the DHIS by facilities		22 (28.21)	15 (19.23)	27 (34.62)	14 (17.95)
		• Frequency of analysis of statistics for decisionmaking by facility staff		19 (24.36)	14 (17.95)	24 (30.77)	21 (26.92)
		• Frequency of feedback reports to facilities by		25 (32.05)	9 (11.54)	21 (26.92)	23 (29.49)

		• Frequency of use of health activity monitoring mechanisms		27 (34.62)	10 (12.82)	18 (23.08)	23 (29.49)
		• Frequency of submission of reports to the DHIS by traditional healers		25 (32.05)	13 (16.67)	16 (20.51)	24 (30.77)

CR, community representative; DHIS, District Health Information System; DNK, do not know; FBO, faith-based organisation; HM, health manager; n, number; Neg, negative; NGO, non-governmental organisation; NN, not negative; SN, somewhat negative; VN, very negative

There were also statistically significant improvements in the median scores of the programme reporting units in respect of the internal processes perspective which improved from 15.4% in the first year to 23.1% in the second year (*p*=0.0172); the finance perspective which improved from 25.0% in the first year to 75.0% in the second year (*p*=0.0174); and overall leadership (Table 3) which improved from 12.0% in the first year to 32.0% in the second year (*p*=0.0135). However, the median BSC scores of the programme reporting units in respect to the customer perspective remained constant at 25.0% in the first and second years. Although the median scores for the organisational capacity perspective improved from zero in the first year to 25.0% in the second year, the difference was not statistically significant (*p*=0.0884).

At the district level, the median BSC scores for the customer perspective decreased from 50.0% in the first year to 25.0% in the second year, although this difference was not statistically significant (*p*=0.3242). Likewise concerning, and again acknowledging that this was not statistically significant (*p*=0.0539), the median score for the organisational capacity perspective declined from 25.0% in the first year to zero in the second year at the district level.

## Discussion

This study described the ‘how-to’ of an intervention to improve leadership/management of public health services using the HSGA approach to improve health service integration and health outcomes by implementation of a ‘whole-system’ intervention in a public health setting where limited resources allocation and a high burden of disease presented major challenges. The overall findings of the study re-assert and emphasise the importance of the leadership/management ‘building block’ in health systems strengthening initiatives.

The relationships between the different components in a public health system are complex and non-linear thereby making implementation of ‘whole-system’ interventions inherently challenging^24^. The necessity to both individually optimise and integrate each health system component became clear as the implementation of the HSGA intervention unfolded. Implementation strategies were thus simultaneously undertaken across multiple dimensions including the six health systems ‘building blocks.’ The guiding principle throughout all improvement initiatives was to reliably provide high-quality, high-value, patient-centred care to the Free State population of whom more than 80% are reliant on the public healthcare system^25^. The study evidences the relative success of the HSGA ‘whole-system’ intervention in bringing about significant improvement in leadership/management in order to advance the integration and outcomes of public health services in the Free State Province. The comparison of the BSC scores over the first (2014/15) and the second (2015/16) years of the study demonstrated medium to large changes on the leadership goal perspectives with an effect change of 0.734 for the overall leadership goal.

From the literature, reported challenges experienced in other health systems strengthening implementation processes include lack of common language, lack of transformation goals, lack of a shared agenda, inappropriate methodologies, weak policies and lack of an embedded evaluation plan^26^. As shown in a systematic analysis of 32 case studies in 24 countries, in public health systems - due to their complexity – barriers to implementation of interventions also include deficits or shortfalls in i) leadership, ii) management and collaboration, iii) funding, iv) capacity, v) data, vi) visibility of the issue being addressed, vii) the evidence-base of the intervention itself, and viii) the context setting^27^. However, according to these authors, each of these eight factors, if effectively managed, can also facilitate success.

The uniqueness and advantage of using the HSGA as a “whole-system.” intervention approach for health service integration, with concomitant application of the BSC as a monitoring and evaluation tool, was the opportunities this presented to identify either deficiencies in individual system and sub-system components or in their interrelatedness to one another and how they influence each other and improve the overall functioning of the public health systems as a ‘whole’.

The participatory approach followed in the design, development^8^ and implementation of the HSGA intervention in the Free State allowed managers, frontline health workers and community stakeholders to ask questions and help think through and understand how the changes would improve leadership/management of patient care or clinical practices and how new approaches would improve the general service offering. The success of the implementation of the HSGA intervention therefore hinged on maintaining a focus on the ‘whole health system:’ the patient, the providers, the facilities, and the policy environment^28^. The participatory design and nature of the study conferred additional strength to it as users were closely engaged in the design and development of the implementation plan, as well as its implementation or operationalisation. Another strength of the HSGA intervention was its focus of the bigger problem of fragmentation of services across the ‘whole public health system’ as opposed to only the lower-level programmes and facilities.

Where it has been shown invaluable in evaluating health systems strengthening interventions at different organisational levels and executing units forming part of health system reform in Zambia^29^, Brazil^30^, Iran^31^, Afghanistan^32^ and Bangladesh^33^, the Free State experience bore further testimony to the usefulness of the BSC performance-monitoring tool to assess (public sector) organisational performance to inform feedback and reporting, as well as progressive implementation of an intervention. Such an approach remains unusual in a regulated public sector environment where business principles in structuring the monitoring and evaluation activities are generally regulated and overseen by provincial treasuries and may not generally be based on business and management principles.

A limitation of this study is that the first author was the Member of the Executive Council (MEC) and the second and fifth authors held co-appointments with the FSDoH during the data gathering. However, social desirability bias was countered by exclusion of the political executive leader during data-gathering and when investigations and interviews were conducted by the DCSTs. The inclusion of co-authors who are not employees of the FSDoH in the analysis and interpretation of the data further contributed to the objectivity of the research.

A recommendation for future research emanating from the current study is to measure and analyse the effects of the intervention on the individual components or building blocks of the Free State public health system. It could be hypothesised that improvements across all the individual components would have resulted due to the improvements in leadership/management and emerging synergies in an overall approach to work together in achieving ‘whole-system’ improvement.

## Conclusion

This paper described the participatory processes that were followed in implementing a system-wide health systems strengthening intervention, formalised into official policy, to improve leadership/management of provincial public healthcare services in the Free State Province. A ‘whole-system’ approach which allowed for improving not only the performance of individual sub-systems, but also their interaction in the process to produce good public health outcomes, was followed. Important improvements in leadership/management were evidenced as a result of implementation of the HSGA intervention.

## Figures and Tables

**Table 6:** Views related to Goal 7 Referral and whole-system interventions

			Always n (%)	Mostly n (%)	Seldom n (%)	Never n (%)	DNK n (%)
**HMs** **(n=147)**	**Referral**	• Processes to refer patients to other facilities in place	93 (64.14)	18 (12.41)	2 (1.38)	0	32 (22.07)
		• Referral notes (from a lower to a higher level) in place	85 (58.62)	23 (15.86)	5 (3.45)	1 (0.69)	31 (21.38)
		• Referral feedback reports (from higher level back to lower level) in place	33 (22.76)	24 (16.55)	44 (30.34)	15 (10.34)	29 (20.00)
		• Ambulance referral/dispatch systems in place	64 (44.14)	41 (28.28)	7 (4.83)	2 (1.38)	31 (21.38)
		• Waiting times in healthcare facilities monitored	80 (55.17)	28 (19.31)	5 (3.45)	1 (0.69)	31 (21.38)
		• Agreements on referral of patients from traditional healers in place	15 (10.34)	7 (4.83)	6 (4.14)	34 (23.45)	83 (57.24)
		• Agreements on referral of patients from NGOs in place	23 (15.86)	19 (13.10)	16 (11.03)	14 (9.66)	73 (50.34)

**CRs** **(n=78)**	**Referral**	• Processes to refer patients to other facilities in place	38 (48.72)	10 (12.82)	10 (12.82)	8 (10.26)	12 (15.38)
		• Referral notes (from a lower to a higher level) in place	41 (52.56)	10 (12.82)	9 (11.54)	4 (5.13)	14 (17.95)
		• Referral feedback reports (from higher level back to lower level) in place	34 (43.59)	14 (17.95)	9 (11.54)	7 (8.97)	14 (17.95)
		• Ambulance referral/dispatch systems in place	36 (46.15)	8 (10.26)	15 (19.23)	9 (11.54)	10 (12.82)
		• Waiting times in healthcare facilities monitored	33 (42.31)	10 (12.82)	11 (14.10)	11 (14.10)	13 (16.67)
		• Agreements on referral of patients from traditional healers and NGOs in place	26 (33.33)	9 (11.54)	10 (12.82)	11 (14.10)	22 (28.21)

					Yes n (%)	No n (%)	DNK n (%)

**HMs** **(n=147)**	**Whole-** **system** **interventions**	• HSGA intervention contributed to integrating health service delivery			65 (44.22)	18 (12.24)	64 (43.54)
		• BSC performance-monitoring tool contributed to integrating health service delivery			60 (40.82)	23 (15.65)	64 (43.54)
		• HSGA intervention contributed to improving health outcomes			61 (41.50)	17 (11.56)	69 (46.94)
		• BSC performance-monitoring tool contributed to improving health outcomes			60 (40.82)	23 (15.65)	64 (43.54)
		• HSGA intervention contributed to integrating health service delivery			53 (67.95)	9 (11.54)	16 (20.51)
		• BSC performance-monitoring tool contributed to integrating health service delivery			39 (66.10)	7 (11.86)	32 (22.03)

**CRs** **(n=78)**	**Whole-** **system** **interventions**	• HSGA intervention contributed to integrating health service delivery			65 (44.22)	18 (12.24)	64 (43.54)
		• BSC performance-monitoring tool contributed to integrating health service delivery			60 (40.82)	23 (15.65)	64 (43.54)
		• HSGA intervention contributed to improving health outcomes			61 (41.50)	17 (11.56)	69 (46.94)
		• BSC performance-monitoring tool contributed to improving health outcomes			60 (40.82)	23 (15.65)	64 (43.54)

		• HSGA intervention contributed to integrating health service delivery			53 (67.9	9 (11.54)	16 (20.51)
		• BSC performance-monitoring tool contributed to integrating health service delivery			39 (66.10)	7 (11.86)	32 (22.03)

BSC, Balanced Scorecard; CR, community representative; DNK, do not know; HM, health manager; HSGA, Health System Governance and Accountability; n, number; NGO, non-governmental organisation
